# Effusive-constrictive pericarditis as first manifestation of late-onset systemic lupus erythematosus: an atypical case with grave prognosis

**DOI:** 10.1186/s43044-023-00353-6

**Published:** 2023-04-20

**Authors:** I. Dewa Gde Dwi Sumajaya, I. Putu Hendri Aryadi, I. Made Eryana

**Affiliations:** 1 Cardiology Department, Dharma Kerti Hospital, Bali, 82113 Indonesia; 2 Emergency Department, Dharma Kerti Hospital, Bali, 82113 Indonesia

**Keywords:** Pericardial effusion, Constrictive pericarditis, Systemic lupus erythematosus, Late-onset

## Abstract

**Background:**

Systemic lupus erythematosus (SLE) is a multisystem autoimmune disease that has a great diversity of clinical presentations and occurs mostly in young women. However, late-onset SLE does exist and seldom presents with an atypical case, including pericardial effusion (PE).

**Case presentation:**

A 64 years old Asian woman presented with weakness all over the body and slight breathlessness for the past 2 days before the hospital admission. Her initial vital signs are 80/50 mmHg for blood pressure and a respiration rate of 24 breaths/min. Rhonchi were heard on the left lung and pitting edema on both legs. No evidence of any skin rash. Laboratory examination displayed anemia, hematocrit decrement, and azotemia. A 12-lead ECG demonstrated left-axis deviation with low voltage (Fig. 1). Chest X-ray showed left massive pleural effusion (Fig. 2). Transthoracic echocardiography revealed biatrial enlargement, normal EF 60%, diastolic dysfunction grade II, and thickening of the pericardium with mild circumferential PE corresponding with effusive-constrictive pericarditis (Fig. 3). The patient also brought CT angiography and cardiac MRI result, which confirmed pericarditis with PE. Treatment was initiated in ICU with fluid resuscitation of normal saline. The patient’s routine oral treatments, including furosemide, ramipril, colchicine, and bisoprolol, were carried on. An autoimmune workup was performed by a cardiologist and demonstrated an elevation in antinuclear antibody/ANA (IF) of 1:100, which finally unveiled a diagnosis of SLE. Pericardial effusion is one critical condition to consider, despite it being an uncommon presentation in late-onset SLE. Mild pericarditis in an SLE case can be treated with corticosteroid administration. Colchicine also has been found to reduce the risk of pericarditis recurrence. However, an atypical presentation from this case led to a slightly delayed treatment that escalated the morbidity and mortality risk. The patient had a sudden cardiac arrest and passed away 3 days after being treated.

**Fig. 1:**
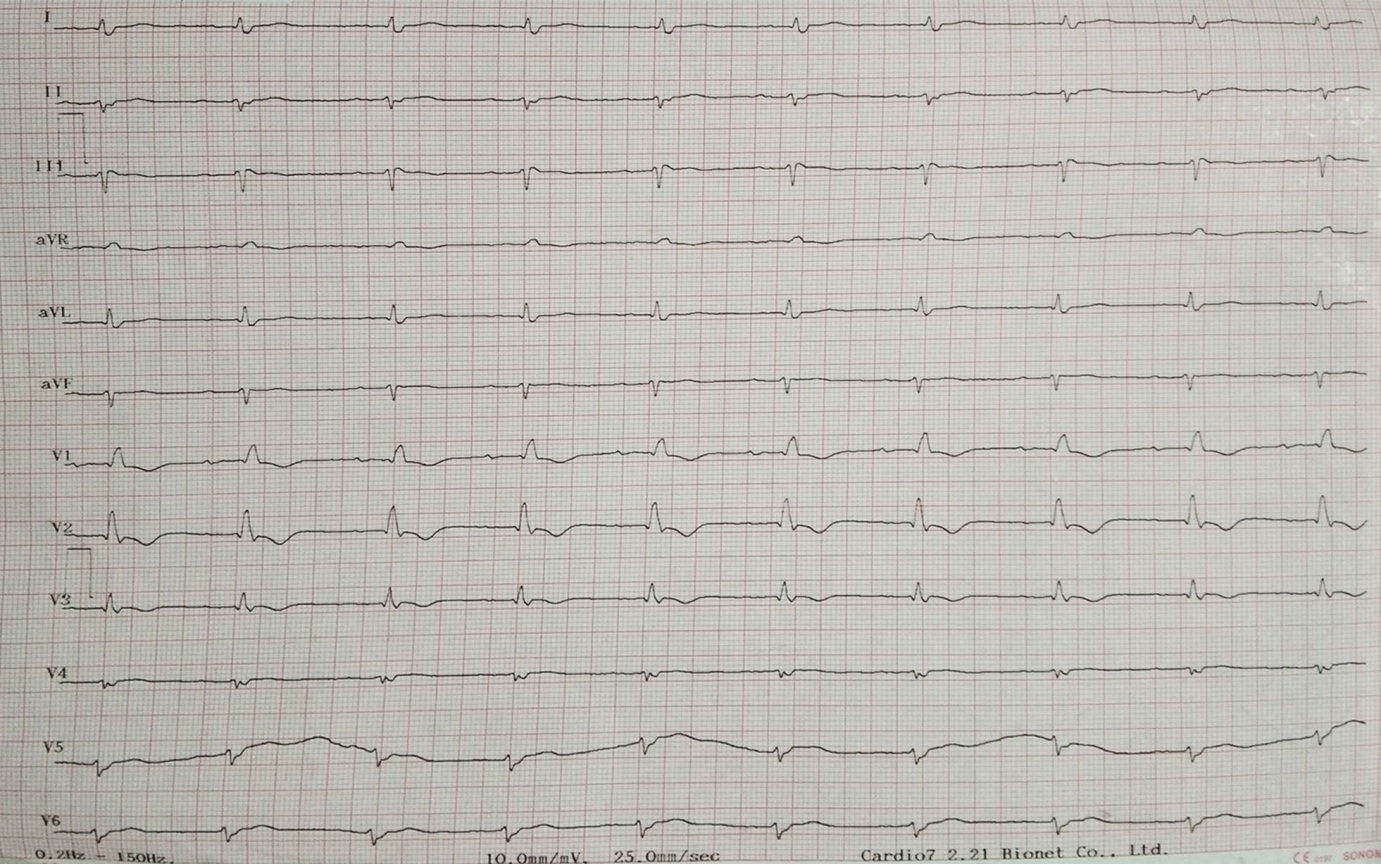
Initial electrocardiogram demonstrated left-axis deviation, low voltage QRS complex and T-wave inversion on lead V1–V3

**Fig. 2:**
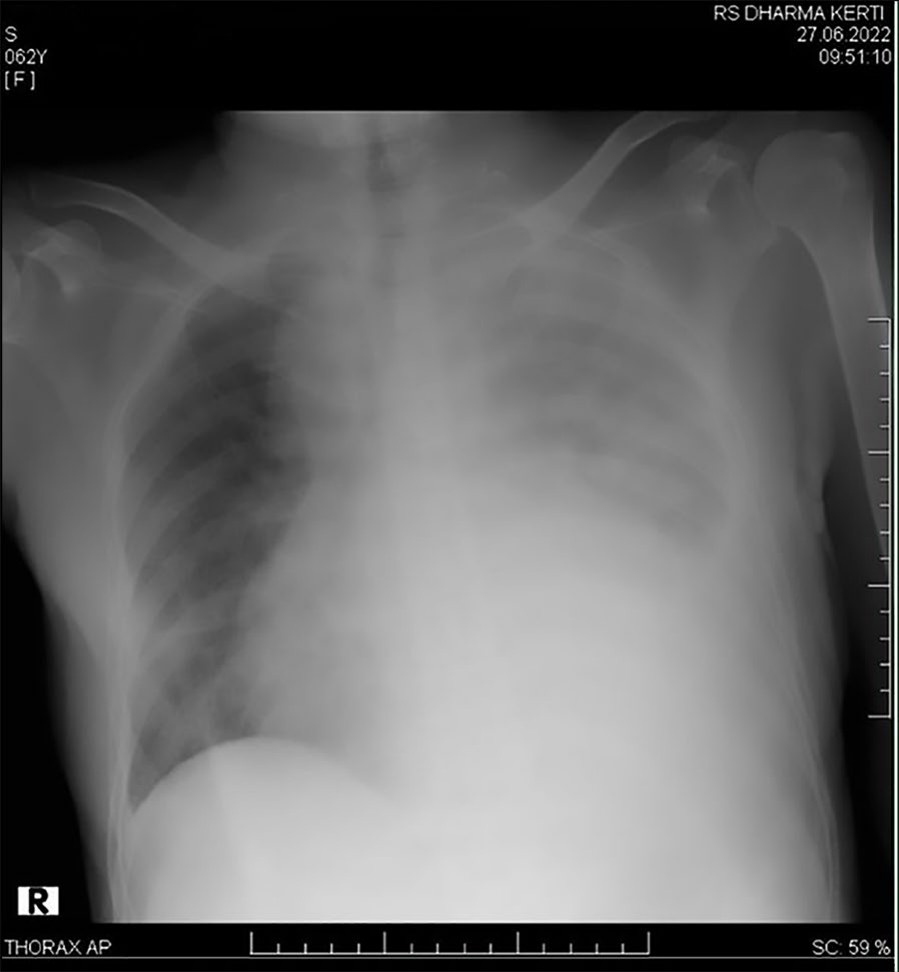
Chest radiograph showed left massive pleural effusion

**Fig. 3:**
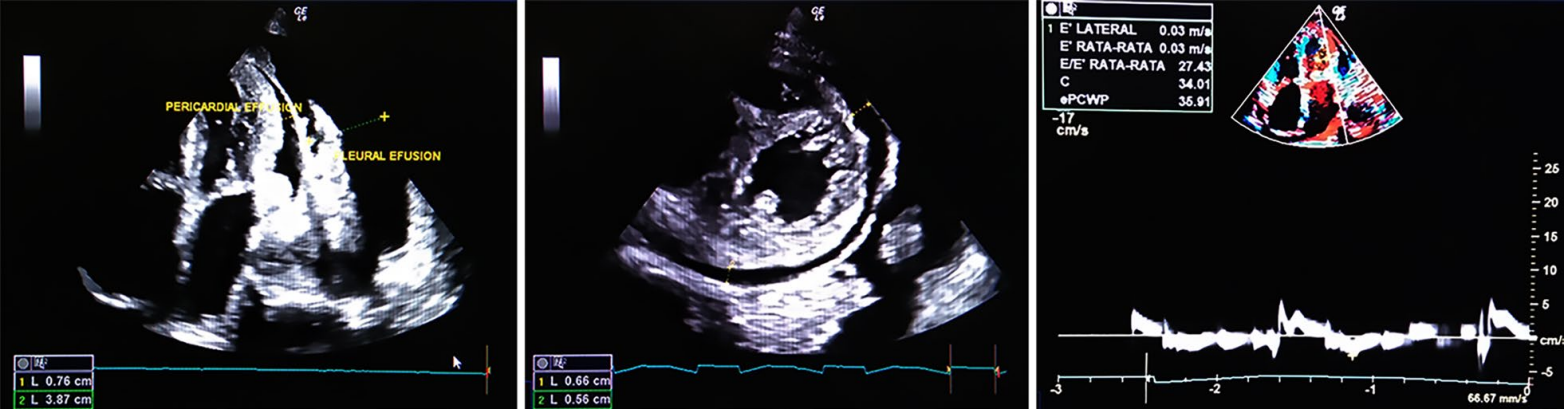
Transthoracic echocardiogram displayed increased left ventricular filling pressure with diastolic dysfunction grade III, mild circumferential pericardial effusion with adjacent pleural effusion

**Conclusions:**

Atypical presentation during late-onset SLE, mainly in the form of pericardial effusion even constrictive pericarditis, should be taken into a consideration since they are a scarce feature in SLE patients. Swift recognition and prompt treatment are important for the optimal outcome.

## Background

Systemic lupus erythematosus (SLE) is a multisystem autoimmune disease that has a great diversity of clinical presentations [1, 2]. As well as most autoimmune diseases, SLE rarely occurs in the elderly. It predominantly affects women during childbearing age and declines right after menopause [1]. However, there is still a probability of 2–20% of SLE cases take place in subjects older than 50 years old, and seldom present with atypical features, including pericarditis and pericardial effusion (PE) [2]. We herein report a case of effusive-constrictive pericarditis as the first manifestation leading to the diagnosis of a late-onset SLE.

## Case presentation

A 64 years old Asian woman presented to the emergency department with weakness all over the body, nausea, and slight breathlessness in the past 2 days before the hospital admission. She had a past medical history of chronic heart failure, pericardial effusion, pleural effusion, non-hemorrhagic stroke, and dyspepsia syndrome. Her initial vital signs were blood pressure of 80/50 mmHg, regular pulse of 65 beats/min, the respiration rate of 24 breaths/min, the temperature of 36.0 °C, and oxygen saturation of 97% on room air. Physical examination disclosed rhonchi on the left lung, distended abdomen, and pitting edema on both legs. No evidence of any rashes or skin lesions was exist. Her laboratory examination displayed anemia (hemoglobin of 9.9 g/dL), decrement of hematocrit, and azotemia (blood urea nitrogen of 134.8 mg/dL, serum creatinine of 2.2 mg/dL). Initial twelve-lead ECG demonstrated left-axis deviation, low voltage QRS complex, and T-wave inversion on lead V1–V3 (Fig. 1). Chest radiograph presented left massive pleural effusion (Fig. 2). Transthoracic echocardiography revealed left and right atrium dilatation, left ventricular concentric hypertrophy, diastolic dysfunction grade III with increased filling pressure, normal ejection fraction 60%, hyperechoic at pericardium with mild circumferential pericardial effusion, and noted left pleural effusion (Fig. 3). Initial diagnosis of chronic heart failure due to suspected infiltrative restrictive cardiomyopathy with preserved ejection fraction (EF) NYHA III, mild pericardial effusion, left massive pleural effusion, and acute kidney injury due to the pre-renal cause were declared. The patient also brought the previous CT angiography and cardiac MRI result, which confirmed pericarditis with pericardial effusion (Table 1).

**Table 1:** Summary of investigations

Investigation	Result
Complete blood count	RBC depleted at 2.91 × 10^6^/mL
	HGB depleted at 9.4 g/dL
	HCT depleted at 27.3%
Kidney Function	BUN elevated at 138.5 mg/dL
	Creatinine elevated at 2.0 mg/dL
Albumin	3.5 g/dL
Electrolyte	K^+^ slight decreased at 3.1 mEg/L
Autoimmune	
ANA (IF)	Speckled pattern with a titer of 1:100
Imaging	CXR:
	Suggestive right pleuropneumonia
	Left massive pleural effusion
	Echocardiogram:
	Circumferential pericardial effusion (Mild)
	Decreased right ventricular systolic function
	Left ventricular diastolic dysfunction grade III

*ANA* antinuclear antibodies, *BUN* blood urea nitrogen, *CXR* chest X‐ray, *HCT* hematocrit, *HGB* hemoglobin, *IF* indirect immunofluorescence, *RBC* red blood cell

Treatment for hypotension was initiated with fluid resuscitation of 300 mL of normal saline. The patient’s routine oral treatments, including furosemide, ramipril, colchicine, sildenafil, bisoprolol, and lansoprazole, were carried on. The patient was then transferred to the ICU and was given supportive therapy, meanwhile, the etiology was still vague. An autoimmune workup was performed with the consideration of serositis, hematologic, and renal function disturbance. It demonstrated an elevated indirect immunofluorescent antinuclear antibody/ANA (IF) of 1:100 with a speckled pattern, which finally unveiled a possible diagnosis of SLE. The patient’s condition had been getting better after three days of in-hospital treatment. Unfortunately, during the preparation for hospital discharge, the patient had a sudden cardiac arrest and passed away.

The onset of SLE in patients over 50 years old is very infrequent [1]. Several studies have examined differences between early-onset SLE and late-onset SLE (over the age of 50) in terms of their clinical manifestations. Serositis and cytopenia are more frequently seen in late-onset SLE patients [3]. On the other hand, dermatological symptoms, such as malar rash and photosensitivity, are less frequent (40.0% and 36.7%, respectively) [2, 3]. This fact is also in-line with our patient. Although no skin rash was found, this elderly patient presented with PE, severe serositis, and a positive ANA test. Therefore, through the calculation of the 2019 EULAR/ACR score, which was 15, the diagnosis of SLE was disclosed. Pericardial effusion is one critical condition to consider in this patient, despite it being an uncommon presentation in late-onset SLE.

Numerous cardiac diseases can take place in connective tissue disorders, as well as in SLE. Although SLE can affect all three heart layers, the most commonly documented cardiac manifestation is pericarditis [4]. A variety of echocardiographic abnormalities were found among SLE patients without any clinically obvious heart disease, including valvular lesions (47.5%), pericardial effusion (13.6%), pericardial thickening (6.8%) and impaired systolic function (3.4%) [5].

Pericarditis itself is mediated by immune complexes and complement system activation [6]. Several antibodies, involving IgG, IgM, IgA, and complement C3 have been detected in the pericardium along its inflammatory process. The prolonged and recurrent effusiveness or fibrous changes during pericardial involvement will progress to the development of constrictive pericarditis [6]. Constrictive pericarditis (CP) refers to a fibrotic progression of the pericardium secondary to an inflammatory process. The pericardium loses its elasticity, becomes stiff and scarred, and eventually fails to distend during diastole. It precedes an impaired right ventricular filling process and sooner or later will be followed by the reduction in left ventricular and stroke volume due to interventricular septum bulging into the left ventricle [7].

The clinical manifestation of pericarditis among SLE patients is the same as the classic presentation of acute pericarditis, including fatigue, dyspnea with typical positional-dependent substernal pleuritic chest pain/orthopnea (aggravated by laying down in a supine position) [8], paroxysmal nocturnal dyspnea, peripheral edema, even ascites. The vital clinical signs are elevated jugular venous pressure (JVP) and a drop of systolic blood pressure of more than 10 mmHg during inspiration (*pulsus paradoxus*) [7, 9]. Furthermore, from physical examination, there can be revealed decreased or muffled heart sounds and pericardial rub [8]. Electrocardiogram commonly displays non-specific T-wave changes to transient ST changes, but in some cases, may show diffusely elevated ST-segments with peaked T-waves [8]. A transthoracic echocardiogram is the standard imaging modality for pericardial effusion, which can reveal the thickening of the pericardium and the amount of effusion, even in the mild level [10]. The accumulation rate of effusion which should be monitored routinely using an echocardiogram is the most powerful predictor of outcome in patients with pericardial effusion [11, 12]. The aspiration and analyzation of pericardial fluid, if present, may assist in some cases with uncertain etiology [9].

Alternative diagnoses should be cautiously considered among patients with the suspicion of pericarditis. There are some other causes of pericarditis that can be considered besides autoimmune, including infection of viral (Epstein-Barr, Adenovirus, and Coxsackie virus), bacterial (Tuberculosis and Whipple’s disease), fungal, or parasitic; neoplasm and paraneoplastic syndromes; metabolic triggers (hypothyroidism and uremia); even radiation and trauma causes [10]. This patient did not show any tendencies to infectious causes, since there were no signs and symptoms of fever, and normal value in all immune-related hematologic profiles. Neoplasm, metabolic, radiation, or trauma-related causes were excluded due to no history and adequate evidence.

Pericardial effusions in SLE are usually mild and rarely compromise the patient’ hemodynamics [8]. Mild pericarditis in an SLE flare can be treated with corticosteroid administration (intramuscular triamcinolone injection or oral methylprednisolone) [13], meanwhile, the severe one should be treated more aggressively with an intravenous bolus of methylprednisolone [8]. It should be administered swiftly after the exclusion of infections [4]. Intravenous immunoglobulin (IVIG), methotrexate, and steroid-sparing agents, such as azathioprine and mycophenolate mofetil, may be necessitated in recurrent pericarditis secondary to SLE [8, 14, 15]. Moreover, a meta-analysis study has confirmed the effect of colchicine in reducing the risk of pericarditis recurrence [16]. In this case, the patient was administered to ICU and given supportive therapy. However, an atypical presentation from this case led to a slightly delayed treatment which escalated the morbidity and mortality risk.

## Conclusions

Patients with SLE sometimes show atypical clinical features as their first manifestation, which makes it harder to formulate a precise diagnosis, moreover in an infrequent late-onset SLE. Although pericardial effusion and constrictive pericarditis are not-so-scarce findings in late-onset SLE patients, they are still counted as atypical first presentations which should be given serious consideration. Swift recognition, accurate diagnosis, and prompt treatment are important for the optimal outcomes of this effusive-constrictive pericarditis in late-onset SLE patients.

## Limitations of the report

The differential diagnosis of tuberculosis or pulmonary metastases had yet to be ruled out. The exception to this possibility was based on history and physical examination only, while no further laboratory tests had been carried out to confirm it.

## Data Availability

The datasets used during the current study are available from the corresponding author on reasonable request.

## Abbreviations

CP: Constrictive pericarditis
EF: Ejection fraction
IVIG: Intravenous immunoglobulin
JVP: Jugular venous pressure
PE: Pericardial effusion
SLE: Systemic lupus erythematosus
